# 1-(4-Carb­oxy­phen­yl)-1*H*-imidazol-3-ium chloride dihydrate

**DOI:** 10.1107/S1600536811001838

**Published:** 2011-01-22

**Authors:** Zhi-Peng Yu, Wen-Qian Geng, Lin Kong, Hong-Ping Zhou

**Affiliations:** a Department of Chemistry, Anhui University, Key Laboratory of Functional Inorganic Materials Chemistry, Hefei 230039, People’s Republic of China

## Abstract

In the crystal structure of the title compound, C_10_H_9_N_2_O_2_^+^·Cl^−^·2H_2_O, the components are linked by O—H⋯O, N—H⋯O, O—H⋯Cl and N—H⋯Cl hydrogen bonds. In the cation, the imidazole ring is oriented at a dihedral angle of 13.67 (17)° with respect to the benzene ring. In the crystal, π–π stacking occurs between nearly parallel benzene rings, which are oriented at a dihedral angle of 3.4 (1)°, the centroid–centroid distance being 3.798 (3) Å.

## Related literature

For related imidazole-containing compounds, see: Nyamori & Bala (2008 ▶); Nie *et al.* (2009 ▶).
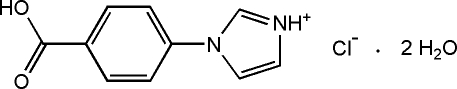


## Experimental

### 

#### Crystal data

C_10_H_9_N_2_O_2_^+^·Cl^−^·2H_2_O
*M* *_r_* = 260.67
Orthorhombic, 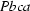

*a* = 7.427 (5) Å
*b* = 17.708 (5) Å
*c* = 18.748 (5) Å
*V* = 2465.7 (19) Å^3^
*Z* = 8
Mo *K*α radiation
μ = 0.32 mm^−1^
*T* = 298 K
0.30 × 0.20 × 0.20 mm

#### Data collection

Bruker SMART CCD area-detector diffractometer
Absorption correction: multi-scan (*SADABS*; Bruker, 2001 ▶) *T*_min_ = 0.912, *T*_max_ = 0.940
14555 measured reflections
2165 independent reflections
1515 reflections with *I* > 2σ(*I*)
*R*_int_ = 0.033

#### Refinement

*R*[*F* ^2^ > 2σ(*F* ^2^)] = 0.040
*wR*(*F* ^2^) = 0.146
*S* = 0.96
2165 reflections
175 parameters
H atoms treated by a mixture of independent and constrained refinement
Δρ_max_ = 0.20 e Å^−3^
Δρ_min_ = −0.32 e Å^−3^

### 

Data collection: *SMART* (Bruker, 2007 ▶); cell refinement: *SAINT* (Bruker, 2007 ▶); data reduction: *SAINT*; program(s) used to solve structure: *SHELXTL* (Sheldrick, 2008 ▶); program(s) used to refine structure: *SHELXTL*; molecular graphics: *SHELXTL*; software used to prepare material for publication: *SHELXTL*.

## Supplementary Material

Crystal structure: contains datablocks I, global. DOI: 10.1107/S1600536811001838/xu5134sup1.cif

Structure factors: contains datablocks I. DOI: 10.1107/S1600536811001838/xu5134Isup2.hkl

Additional supplementary materials:  crystallographic information; 3D view; checkCIF report

## Figures and Tables

**Table 1:** Hydrogen-bond geometry (Å, °)

*D*—H⋯*A*	*D*—H	H⋯*A*	*D*⋯*A*	*D*—H⋯*A*
N2—H2⋯O1^i^	0.86	2.21	2.822 (3)	128
N2—H2⋯Cl1^ii^	0.86	2.62	3.231 (2)	129
O2—H7⋯O4^iii^	0.82	1.77	2.594 (3)	177
O3—H10⋯Cl1^iv^	0.83 (4)	2.36 (4)	3.150 (3)	159 (3)
O3—H11⋯Cl1^v^	0.94 (5)	2.22 (5)	3.141 (3)	168 (3)
O4—H12⋯O3	0.76 (4)	1.99 (4)	2.718 (4)	162 (4)
O4—H13⋯Cl1	0.86 (4)	2.22 (4)	3.066 (4)	172 (3)

Symmetry codes: (i) 
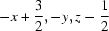
; (ii) 
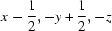
; (iii) 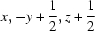; (iv) 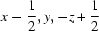; (v) 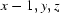.

**Fig. 1.**
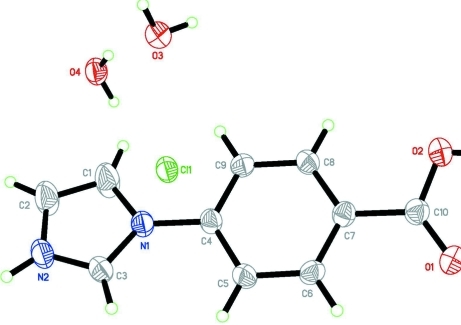
The molecular structure of the title molecule(I) showing 30% probability displacement ellipsoids.

**Fig. 2.**
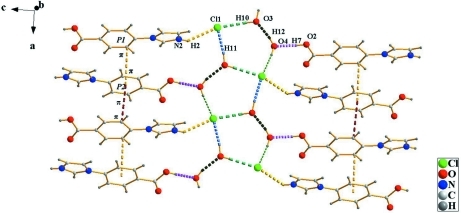
The H-bond and weak π–π interaction diagram of the title molecule(I).

## supplementary crystallographic information

### Comment

Imidazol - based materials had been investigated for their electrical and optical properties. Beside, the introduction about the structure of its complex has been reported (Nie *et al.*, 2009). The title molecule that we has designed and synthesized is a good intermediate and penetratingly investigated. In the title molecule(I) (Fig.1), the bond lengths and angles show normal values. The imidazole ring is twisted out of the plane of the center benzene ring at a dihedral angle of 13.67 (17)°. In the title molecule (I) (Fig.2), the neighboring molecules connect through O—H···O, N—H···O, O—H···Cl and N—H···Cl hydrogen bonds (Table 1). π–π stacking is observed between nearly parallel benzene rings, the centroids distance being 3.798 (3) Å.

### Experimental

A 150 ml round-bottom flask was charged with a magnetic stirrer and a reflux condenser, iminazole (44 mmol), K_2_CO_3_ (6.00 g, 43 mmol), 30 ml DMSO and a little Aliquat 336 were added. 4-Fluorobenzaldehyde (4.5 ml, 42 mmol) was added dropwise to the mixture at 363 K and stirred for 15 min. Then the reaction mixture was refluxed for 24 h at 353 K, cooled to room temperature, poured into 150 ml ice-water and filtered. The primrose yellow crude product was obtained, washed with distilled water, and dried *in vacuo* at room temperature, then purified by recrystallization with ethyl acetate to give the desired analytical pure intermediate products. Intermediate product (12.5 mmol) and 15 ml 20% (wt) NaOH (aq) were added to a round-bottom flask equipped with a magnetic stirrer and a reflux condenser at 333 K for 30 min. Then AgNO_3_ (4.00 g, 24 mmol) was added to the mixture group by group. The reaction mixture was refluxed for 24 h at 333 K, cooled to room temperature and filtered. Excessive HCl (1 *M*) was added to the filtrate and adjust pH to 2, a great deal of sediments were obtained and then filtered. The crude product was recrystallized from ethanol-water solution.

### Refinement

Water H atoms were located in a difference Fourier map and refined isotropically. Other H atoms were placed in geometrically idealized positions and constrained to ride on their parent atoms, with O—H = 0.82 and C—H = 0.93 Å, U_iso_(H) = 1.5U_eq_(O) and 1.2U_eq_(C).

### Crystal data

**Table:**

C_10_H_9_N_2_O_2_^+^·Cl^−^·2H_2_O	*F*(000) = 1088
*M**_r_* = 260.67	*D*_x_ = 1.404 Mg m^−^^3^
Orthorhombic, *P**b**c**a*	Mo *K*α radiation, λ = 0.71069 Å
Hall symbol: -P 2ac 2ab	Cell parameters from 3445 reflections
*a* = 7.427 (5) Å	θ = 2.3–21.7°
*b* = 17.708 (5) Å	µ = 0.32 mm^−^^1^
*c* = 18.748 (5) Å	*T* = 298 K
*V* = 2465.7 (19) Å^3^	Block, yellow
*Z* = 8	0.30 × 0.20 × 0.20 mm

### Data collection

**Table:**

Bruker SMART CCD area-detector diffractometer	2165 independent reflections
Radiation source: fine-focus sealed tube	1515 reflections with *I* > 2σ(*I*)
graphite	*R*_int_ = 0.033
φ and ω scans	θ_max_ = 25.0°, θ_min_ = 2.3°
Absorption correction: multi-scan (*SADABS*; Bruker, 2001)	*h* = −8→7
*T*_min_ = 0.912, *T*_max_ = 0.940	*k* = −21→16
14555 measured reflections	*l* = −17→22

### Refinement

**Table:**

Refinement on *F*^2^	Primary atom site location: structure-invariant direct methods
Least-squares matrix: full	Secondary atom site location: difference Fourier map
*R*[*F*^2^ > 2σ(*F*^2^)] = 0.040	Hydrogen site location: inferred from neighbouring sites
*wR*(*F*^2^) = 0.146	H atoms treated by a mixture of independent and constrained refinement
*S* = 0.96	*w* = 1/[σ^2^(*F*_o_^2^) + (0.1*P*)^2^ + 0.3*P*] where *P* = (*F*_o_^2^ + 2*F*_c_^2^)/3
2165 reflections	(Δ/σ)_max_ < 0.001
175 parameters	Δρ_max_ = 0.20 e Å^−^^3^
0 restraints	Δρ_min_ = −0.32 e Å^−^^3^

### Special details

**Table:**

Geometry. All e.s.d.'s (except the e.s.d. in the dihedral angle between two l.s. planes) are estimated using the full covariance matrix. The cell e.s.d.'s are taken into account individually in the estimation of e.s.d.'s in distances, angles and torsion angles; correlations between e.s.d.'s in cell parameters are only used when they are defined by crystal symmetry. An approximate (isotropic) treatment of cell e.s.d.'s is used for estimating e.s.d.'s involving l.s. planes.
Refinement. Refinement of *F*^2^ against ALL reflections. The weighted *R*-factor *wR* and goodness of fit *S* are based on *F*^2^, conventional *R*-factors *R* are based on *F*, with *F* set to zero for negative *F*^2^. The threshold expression of *F*^2^ > σ(*F*^2^) is used only for calculating *R*-factors(gt) *etc*. and is not relevant to the choice of reflections for refinement. *R*-factors based on *F*^2^ are statistically about twice as large as those based on *F*, and *R*- factors based on ALL data will be even larger.

### Fractional atomic coordinates and isotropic or equivalent isotropic displacement parameters (Å^2^)

**Table:**

	*x*	*y*	*z*	*U*_iso_*/*U*_eq_	
Cl1	0.99581 (9)	0.35765 (4)	0.14987 (4)	0.0637 (3)	
O3	0.4071 (3)	0.34344 (12)	0.18640 (13)	0.0661 (6)	
N1	0.6552 (3)	0.13527 (10)	0.12446 (10)	0.0467 (5)	
C4	0.6689 (3)	0.11991 (13)	0.19963 (12)	0.0423 (6)	
O4	0.6325 (4)	0.41591 (12)	0.09501 (10)	0.0628 (6)	
O2	0.6026 (3)	0.11860 (11)	0.46109 (9)	0.0678 (6)	
H7	0.6154	0.1068	0.5031	0.102*	
O1	0.7751 (3)	0.01818 (11)	0.44419 (10)	0.0717 (6)	
C10	0.6934 (3)	0.07130 (14)	0.42051 (13)	0.0484 (6)	
C6	0.7468 (3)	0.03739 (12)	0.29454 (12)	0.0473 (6)	
H6	0.7936	−0.0083	0.3105	0.057*	
C7	0.6859 (3)	0.09017 (12)	0.34359 (12)	0.0436 (6)	
C5	0.7388 (3)	0.05180 (13)	0.22262 (12)	0.0481 (6)	
H5	0.7798	0.0163	0.1899	0.058*	
C8	0.6198 (3)	0.15821 (13)	0.31931 (13)	0.0483 (6)	
H8	0.5805	0.1941	0.3520	0.058*	
C9	0.6110 (3)	0.17379 (12)	0.24755 (13)	0.0469 (6)	
H9	0.5667	0.2199	0.2316	0.056*	
N2	0.6424 (3)	0.12032 (12)	0.01107 (11)	0.0572 (6)	
H2	0.6454	0.0992	−0.0302	0.069*	
C3	0.6691 (3)	0.08596 (14)	0.07173 (13)	0.0532 (7)	
H3	0.6941	0.0348	0.0771	0.064*	
C2	0.6095 (6)	0.19392 (17)	0.02329 (16)	0.0848 (11)	
C1	0.6184 (6)	0.20415 (17)	0.09362 (16)	0.0934 (12)	
H1	0.6026	0.2496	0.1176	0.112*	
H12	0.554 (5)	0.402 (2)	0.117 (2)	0.079 (13)*	
H13	0.734 (5)	0.3965 (18)	0.1065 (16)	0.076 (11)*	
H11	0.283 (6)	0.341 (2)	0.179 (2)	0.118 (15)*	
H10	0.421 (6)	0.359 (2)	0.228 (2)	0.112 (15)*	
H4	0.597 (5)	0.230 (2)	−0.017 (2)	0.112 (12)*	

### Atomic displacement parameters (Å^2^)

**Table:**

	*U*^11^	*U*^22^	*U*^33^	*U*^12^	*U*^13^	*U*^23^
Cl1	0.0640 (5)	0.0782 (5)	0.0489 (5)	−0.0013 (3)	0.0008 (3)	−0.0005 (3)
O3	0.0661 (15)	0.0778 (14)	0.0545 (14)	0.0097 (11)	0.0031 (11)	−0.0011 (11)
N1	0.0583 (12)	0.0435 (11)	0.0383 (11)	−0.0004 (9)	−0.0028 (9)	−0.0008 (9)
C4	0.0480 (13)	0.0421 (12)	0.0370 (13)	−0.0030 (10)	−0.0038 (10)	−0.0021 (10)
O4	0.0735 (15)	0.0696 (13)	0.0452 (12)	0.0004 (13)	0.0000 (12)	0.0038 (9)
O2	0.0905 (15)	0.0737 (12)	0.0393 (10)	0.0220 (11)	0.0038 (10)	0.0017 (10)
O1	0.0959 (15)	0.0695 (12)	0.0497 (11)	0.0272 (12)	−0.0011 (10)	0.0107 (9)
C10	0.0514 (14)	0.0493 (13)	0.0444 (14)	0.0007 (12)	−0.0044 (12)	−0.0011 (12)
C6	0.0571 (14)	0.0351 (11)	0.0497 (14)	0.0043 (11)	−0.0047 (12)	0.0016 (11)
C7	0.0460 (13)	0.0431 (13)	0.0416 (13)	−0.0026 (10)	−0.0050 (10)	0.0009 (10)
C5	0.0623 (15)	0.0406 (13)	0.0413 (13)	0.0032 (11)	0.0000 (12)	−0.0057 (11)
C8	0.0602 (16)	0.0449 (13)	0.0397 (14)	0.0045 (11)	−0.0018 (11)	−0.0059 (10)
C9	0.0583 (15)	0.0374 (11)	0.0450 (14)	0.0064 (11)	−0.0015 (11)	0.0012 (11)
N2	0.0716 (15)	0.0631 (14)	0.0369 (12)	−0.0002 (11)	−0.0019 (10)	−0.0056 (10)
C3	0.0677 (17)	0.0500 (14)	0.0420 (14)	0.0050 (12)	−0.0007 (12)	−0.0075 (12)
C2	0.152 (3)	0.0592 (19)	0.0428 (17)	0.011 (2)	−0.0073 (19)	0.0062 (14)
C1	0.186 (4)	0.0480 (16)	0.0467 (17)	0.017 (2)	−0.010 (2)	0.0026 (13)

### Geometric parameters (Å, °)

**Table:**

O3—H11	0.94 (5)	C6—C7	1.387 (3)
O3—H10	0.83 (4)	C6—H6	0.9300
N1—C3	1.323 (3)	C7—C8	1.379 (3)
N1—C1	1.377 (3)	C5—H5	0.9300
N1—C4	1.439 (3)	C8—C9	1.375 (3)
C4—C9	1.379 (3)	C8—H8	0.9300
C4—C5	1.382 (3)	C9—H9	0.9300
O4—H12	0.76 (4)	N2—C3	1.305 (3)
O4—H13	0.86 (3)	N2—C2	1.346 (4)
O2—C10	1.317 (3)	N2—H2	0.8600
O2—H7	0.8200	C3—H3	0.9300
O1—C10	1.204 (3)	C2—C1	1.333 (4)
C10—C7	1.481 (3)	C2—H4	0.99 (4)
C6—C5	1.373 (3)	C1—H1	0.9300
			
H11—O3—H10	106 (4)	C4—C5—H5	120.5
C3—N1—C1	106.6 (2)	C9—C8—C7	121.0 (2)
C3—N1—C4	127.0 (2)	C9—C8—H8	119.5
C1—N1—C4	126.3 (2)	C7—C8—H8	119.5
C9—C4—C5	121.2 (2)	C8—C9—C4	118.9 (2)
C9—C4—N1	119.0 (2)	C8—C9—H9	120.5
C5—C4—N1	119.8 (2)	C4—C9—H9	120.5
H12—O4—H13	115 (3)	C3—N2—C2	109.3 (2)
C10—O2—H7	109.5	C3—N2—H2	125.3
O1—C10—O2	122.8 (2)	C2—N2—H2	125.3
O1—C10—C7	123.6 (2)	N2—C3—N1	109.4 (2)
O2—C10—C7	113.6 (2)	N2—C3—H3	125.3
C5—C6—C7	120.8 (2)	N1—C3—H3	125.3
C5—C6—H6	119.6	C1—C2—N2	106.9 (3)
C7—C6—H6	119.6	C1—C2—H4	132 (2)
C8—C7—C6	119.1 (2)	N2—C2—H4	121 (2)
C8—C7—C10	122.1 (2)	C2—C1—N1	107.8 (3)
C6—C7—C10	118.8 (2)	C2—C1—H1	126.1
C6—C5—C4	119.0 (2)	N1—C1—H1	126.1
C6—C5—H5	120.5		
			
C3—N1—C4—C9	−165.3 (2)	C6—C7—C8—C9	1.0 (4)
C1—N1—C4—C9	12.2 (4)	C10—C7—C8—C9	−178.7 (2)
C3—N1—C4—C5	14.4 (4)	C7—C8—C9—C4	0.3 (4)
C1—N1—C4—C5	−168.1 (3)	C5—C4—C9—C8	−1.5 (4)
C5—C6—C7—C8	−1.1 (4)	N1—C4—C9—C8	178.3 (2)
C5—C6—C7—C10	178.6 (2)	C2—N2—C3—N1	−0.2 (3)
O1—C10—C7—C8	−167.9 (3)	C1—N1—C3—N2	−0.3 (3)
O2—C10—C7—C8	11.7 (3)	C4—N1—C3—N2	177.6 (2)
O1—C10—C7—C6	12.4 (4)	C3—N2—C2—C1	0.6 (4)
O2—C10—C7—C6	−168.0 (2)	N2—C2—C1—N1	−0.7 (4)
C7—C6—C5—C4	0.0 (4)	C3—N1—C1—C2	0.6 (4)
C9—C4—C5—C6	1.4 (4)	C4—N1—C1—C2	−177.3 (3)
N1—C4—C5—C6	−178.4 (2)		

### Hydrogen-bond geometry (Å, °)

**Table:**

*D*—H···*A*	*D*—H	H···*A*	*D*···*A*	*D*—H···*A*
N2—H2···O1^i^	0.86	2.21	2.822 (3)	128
N2—H2···Cl1^ii^	0.86	2.62	3.231 (2)	129
O2—H7···O4^iii^	0.82	1.77	2.594 (3)	177
O3—H10···Cl1^iv^	0.83 (4)	2.36 (4)	3.150 (3)	159 (3)
O3—H11···Cl1^v^	0.94 (5)	2.22 (5)	3.141 (3)	168 (3)
O4—H12···O3	0.76 (4)	1.99 (4)	2.718 (4)	162 (4)
O4—H13···Cl1	0.86 (4)	2.22 (4)	3.066 (4)	172 (3)

Symmetry codes: (i) −*x*+3/2, −*y*, *z*−1/2; (ii) *x*−1/2, −*y*+1/2, −*z*; (iii) *x*, −*y*+1/2, *z*+1/2; (iv) *x*−1/2, *y*, −*z*+1/2; (v) *x*−1, *y*, *z*.
